# Rotavirus Epidemiology in Bangui, Central African Republic, 2008^1^

**DOI:** 10.3201/eid2007.131839

**Published:** 2014-07

**Authors:** Ionela Gouandijka-Vasilache, Alexandre Manirakiza, Jean Chrysostom Gody, Virginie Banga-Mingo, Odilon Omon Kongombe, Mathew D. Esona, Michael D. Bowen, Diane Waku-Kouomou

**Affiliations:** Institut Pasteur, Bangui, Central African Republic (I. Gouandjika-Vasilache, A. Manirakiza, V. Banga-Mingo);; Complexe Pédiatrique, Bangui (J. Chrysostom Gody);; Village SOS Enfants, Bouar, Central African Republic (O. Omon Kongombe);; US Centers for Disease Control and Prevention, Atlanta, Georgia, USA (M.D. Esona, M.D. Bowen, D. Waku-Kouomou)

**Keywords:** Rotavirus, diarrhea, Bangui, genotyping, Central African Republic, viruses

**To the Editor:** Infection with group A rotavirus is among the leading causes of gastroenteritis in children, especially in sub-Saharan Africa (*1*). Data with regard to the incidence of rotavirus-A disease in the Central African Republic are limited (*2*). To estimate the prevalence of rotavirus-A disease among young children before introduction of rotavirus-A vaccine in Bangui, the capital of the Central African Republic, we performed a prospective study during February–September 2008. The target sample size, based on an expected 20% prevalence of rotavirus diarrhea and a 5% significance level, was 250 cases. All patients were children 0–5 years of age, who were hospitalized for acute diarrhea at the Complexe Pédiatrique, Bangui, the main hospital for children in the Central African Republic, and all had an illness that met the World Health Organization definition of a suspected case of rotavirus-A gastroenteritis (www.who.int/nuvi/surveillance/RV_Case_Defs.pdf). After informed consent and epidemiologic and clinical data had been obtained, a fecal specimen was collected from each child. Samples were transported to the Institut Pasteur de Bangui, where they were tested for rotavirus-A antigen by using the VIKIA Rota-Adeno test, (VIKIA Rota-Adeno; bioMérieux SA, Lyon, France). Results were immediately reported ​​ to the referring physician.

Rotavirus-A G-type (virus protein [VP] 7) and P-type (VP4) genotyping were performed by using previously described 2-step amplification methods (*3*). Extracted double-stranded RNA was denatured at 97°C for 5 min, and VP7 and VP4 were amplified by reverse transcription PCR (RT-PCR) by using consensus primers 9Con1-L/ VP7R (*3*) and Con3/Con2 (*3*), respectively, and the One-Step RT-PCR kit (QIAGEN, Inc., Valencia, CA, USA) according to the manufacturer’s instructions. RT-PCR was conducted by using a GeneAMP PCR System 9700 thermocycler (Applied Biosystems, Foster City, CA, USA) with the following thermocycling profile: 30 min at 42°C; 15 min at 95°C; 35 cycles of 30 s at 94°C, 30 s at 42°C, and 45 s at 72°C; followed by a final 7-min extension at 72°C. G-typing used primer 9Con1-L in combination with primers 9T1–1, 9T1-Dg, 9T-2, 9T-3P, 9T-4, and 9T-9B; P-typing used primer Con3 in combination with primers 1T-1, 1T1-VN, 2T-1, 3T-1, 4T-1, 5T-1, and 1T1-Wa. Genotyping reactions were analyzed by use of electrophoresis on a 3% agarose gel.

Data were analyzed by using Stata 11 software (StataCorp, College Station, TX, USA). Prevalence was expressed in percentages. The χ^2^ test was used to analyze categorical variables and testing by the Yates correction, as appropriate. A 95% confidence interval was calculated, and p​​<0.05 was considered significant. Ethical and administrative permissions were obtained from the National Committee of Ethics of the Central African Republic; the Complexe Pédiatrique, Bangui; and the Central African Republic Government Ministries.

A total of 250 infants and young children with diarrhea (159 male and 91 female, mean age 8.2 months) were enrolled in this study. Results obtained by the VIKIA Rota-Adeno test revealed that 100 (40%) of these children were infected with rotavirus-A, mostly male children (61/100, p˂0.5).The proportions of rotavirus-A infection in children <9 months of age and those ≥10 months of age were 37.3% (62/166) and 45.2% (38/84), respectively, (p = 0.2). Rotavirus-A infections were more prevalent during February–March (67/108, 62.0%) than during April–September (33/142, 23.2%) (p<10^−6^). Because data were collected for only 8 months, annual rotavirus-A prevalence might have been underestimated or overestimated, a possible limitation of the study.

Among the 100 ROTAV-A–positive -patients, 32 samples were randomly selected for genotyping. Among these samples, type G1 predominated (62.8%, 22/32); among P genotypes, type P[8] predominated (50%, 16/32), followed by P[6] (25%, 8/32). The predominant genotypic combination was G1P[8] (43.7%, 14/32) and G1P[6] (25%, 8/32) (Table).

**Table:** Genotyping results for 32 human rotavirus group A–positive samples, Bangui, Central African Republic, 2008

G genotype	P genotype		
	P[4]	P[6]	P[8]
G1	0	8	14
G2	3	0	0
G9	0	0	2
Not typeable	3	0	2

Despite the limitations of a short study period and low number of genotyped strains, this study reports useful information. It reveals that 40% of children hospitalized with acute diarrhea at Complexe Pédiatrique, Bangui, were infected with rotavirus-A during the study period, which included the end of the rotavirus-A gastroenteritis season. Most patients were <9 months of age. These results are similar to those found in the 1980s study at Complexe Pédiatrique, Bangui (*2*), which were that the major serotype/genotype was G1 (71.3%), followed by G2 (15.4%) and G3 (13.3%) (*4*). After 25 years, the predominant circulating genotypes are G1P[8] and G1P[6] along with G2P[4] and G9P[8]. Our study results are similar to those of recent studies conducted in other African countries (*5**–**8*) and confirm results of studies that found that the same genotypes circulated in western Cameroon in 2003, albeit at different percentages (*4*,*9*).

Our study provides relevant data about the genotypes of rotavirus-A from children in the Central African Republic, 25 years after the most recent study (*2*). These data represent baseline information that will help with monitoring for potential changes in genotype prevalence after the introduction of rotavirus-A vaccine in the Central African Republic.
